# Infertility rates following POMB/ACE chemotherapy for male and female germ cell tumours – a retrospective long-term follow-up study

**DOI:** 10.1038/sj.bjc.6601383

**Published:** 2003-11-11

**Authors:** J Gaffan, L Holden, E S Newlands, D Short, S Fuller, R H J Begent, G J S Rustin, M J Seckl

**Affiliations:** 1 Department of Medical Oncology, Charing Cross Campus of Imperial College London, Fulham Palace Rd, London W6 8RF, UK; 2 Department of Oncology, Royal Free and University College Medical School, Rowland Hill Street, London NW3 2PF, UK; 3 Mount Vernon Hospital Cancer Centre, Northwood, Middlesex HA6 2RN, UK

**Keywords:** germ cell tumours, chemotherapy, infertility, cisplatinum

## Abstract

The risk of chemotherapy-induced infertility in male and female germ cell tumour (GCT) survivors is unclear, but may correlate with cisplatin dose. Here, we examine a large series of GCT patients for the effect of chemotherapy on those attempting to have children. Our GCT database was screened for nonseminomatous GCT patients who had (1) received POMB/ACE chemotherapy (cisplatin, vincristine, methotrexate, bleomycin alternating with actinomycin D, cyclophosphamide and etoposide) and (2) stage I male GCT patients who were untreated between 1977 and 1996. Fertility was assessed by questionnaire and medical records. A total of 64 of 153 treated and 35 of 115 untreated men attempted to have children. In all, 28% (18 out of 64) receiving POMB/ACE were unsuccessful. Radiotherapy (six), atrophic remaining testis (one) or prior infertility (three) were implicated in 10 cases, so chemotherapy-induced infertility may have occurred in only 11% (eight out of 64). Strikingly, 26% (nine out of 35) of untreated stage I patients also failed to have children (three had radiotherapy, three prior infertility). Moreover, in treated men, no association was seen between cisplatin dose and infertility. In contrast, radiotherapy significantly increased male infertility (*P*=0.001). Of 28 treated women who attempted to have children, 25% (seven out of 28) were unsuccessful. One previously had infertility and one subsequently had successful IVF so chemotherapy-induced infertility potentially occurred in only 18% (five out of 28) and was not related to cisplatin dose. In conclusion, the risk of chemotherapy-induced infertility is low in both male and female GCT patients and does not clearly correlate with the cumulative cisplatin dose.

Many young patients with cancers, including lymphomas, leukaemias and germ cell tumours (GCTs), are cured with multiagent chemotherapy. Indeed, we have previously reported survival rates of 82% in men and 87.8% in women with GCTs at 5 and 3 years postchemotherapy, respectively (Bower *et al*, 1996, 1997). While short-term toxicities of chemotherapy are well understood, some of the long-term toxicities such as the incidence of infertility are poorly defined with quoted rates as high as 60% in male and 40% in female subjects. This is partly because infertility in patients with GCTs can be associated with multiple variables including emotional distress (Jonker-Pool *et al*, 1997), the primary tumour itself, the initial surgery to remove the affected testis or ovary, any subsequent surgery, radiotherapy as well as with chemotherapy (Schilsky, 1989).

Thus, even before chemotherapy, 22–63% of patients with testicular GCTs have reduced sperm counts at diagnosis (Hartmann *et al*, 1999; Fossa and Kravdal, 2000). Indeed, abnormal sperm counts may be associated with an increased risk of developing GCTs (Jacobsen *et al*, 2000). Orchidectomy alone can result in oligo or azoospermia (Petersen *et al*, 1998) and biopsy of the ‘normal’ contra-lateral testis shows abnormal spermatogenesis in up to 24% of cases (Berthelsen and Skakkebaek, 1983). Subsequent retroperitoneal lymph node dissection (RPLND), if performed, can also induce substantial infertility (Arai *et al*, 1997) although with modern techniques this usually only occurs in 10–15% of cases (Petersen *et al*, 1998). In women, the combined effects of the tumour and surgery to remove ovarian GCTs may result in pelvic scarring, which can contribute to infertility. However, exact figures for pretreatment and surgically induced infertility in women with ovarian GCTs are not available. Radiotherapy, when used to treat male seminomas or its female equivalent, dysgerminomas, can also contribute to infertility. Radiotherapy doses in excess of 6–8 Gy in one fraction to the testes themselves will cause irreversible azoospermia. Similarly, in women, ovarian failure will inevitably result if the ovaries are irradiated. For men, radiotherapy to the retroperitoneal lymph nodes can affect spermatogenesis, even with testicular shielding. This appears to be dose related as 100% of patients receiving <1 Gy recovered spermatogenesis, whereas only 60% recovered at doses >2 Gy in one study (Hansen *et al*, 1990).

During chemotherapy, 96% of men with GCTs become azoospermic (Drasga *et al*, 1983) although most will recover sperm counts following treatment (Lampe *et al*, 1997). Certain chemotherapeutic agents are known to affect fertility more than others. For GCTs, the most common sterilising drug is cisplatin, but in studies of patients with Hodgkin's disease or sarcoma, mustine, busulphan, procarbazine, chlorambucil (>400 mg m^−2^), and cyclophosphamide (>7500 mg m^−2^) were the drugs most likely to sterilise. It has been suggested that the threshold for cisplatin-related infertility is 400 mg m^−2^ (Pont and Albrecht, 1997). Interestingly, cisplatin doses >400 mg m^−2^ have also been shown to correlate with the development of other long-term toxicities in patients with GCTs (Bokemeyer *et al*, 1996; DeSantis *et al*, 1999).

To complicate matters further, previous studies of treatment-induced infertility have used various outcome measures of fertility, including recovery of sperm counts and normal menstrual cycles. However, women with regular periods and men with normal sperm counts can be infertile (Hansen *et al*, 1991), and men with oligospermia can father children (Stephenson *et al*, 1995). Moreover, some patients may be fertile but never wish or attempt to have children. Clearly, the most distressing outcome for the patients is where attempts to have children have failed. It has been argued that since only one-third of patients wish to sire children after treatment for GCTs, the group who try and fail to have children are not representative (Pont and Albrecht, 1997). Nevertheless, infertility is a significant outcome only in patients who wish for children. Provided follow-up times are long enough, the method of assessing chemotherapy-induced infertility by assessing the unfulfilled desire for children should detect all clinically important events.

Here, we investigate rates of infertility (defined as the unfulfilled wish for children) after a specific chemotherapy regime, POMB/ACE (cisplatinum, vincristine, methotrexate, and bleomycin, alternating with actinomycin D, cyclophosphamide, and etoposide) (Newlands *et al*, 1986) in long-term survivors of GCTs. We compare the infertility rates with those seen in our stage I male nonseminomatous germ cell tumours and with previously published series. We also ask whether a relationship can be demonstrated between cumulative dose of cisplatin and the risk of infertility.

## PATIENTS AND METHODS

The male and female GCT computer database at our institute was screened to identify all patients who had received chemotherapy between 1977 and 1996 or had stage I disease and had never received chemotherapy. We identified 437 patients (366 men and 71 women) who had received POMB/ACE, the standard regimen used to treat GCTs at our centre (Newlands *et al*, 1983; Bower *et al*, 1996; Bower *et al*, 1997), and 170 stage I male patients who were untreated on our surveillance programme (Francis *et al*, 2000). Only nonseminomatous germ cell tumours (NSGCT) were included to reduce inclusion of male patients who had been treated with radiotherapy. Moreover, patients who had: (a) sterilising surgery (two men and 19 women); (b) a primary site other than the gonads (23 men); (c) were too young or too old (four men); (d) had ovarian dysgenesis with a 46XY karyotype (one woman); (e) had ovarian ablation with radiotherapy (one woman); or (f) were known to be homosexual (three patients) were excluded from the study. A further 38 patients (all men) (10%) were lost to follow-up and so were also not included. The patients who were too young or too old were aged 88, 64, 64, and 18 years at the time of the questionnaire. In all cases, although a questionnaire was not sent, the patients were known to either have already completed or not started their families.

Questionnaires were sent to all remaining patients enquiring about fertility before and after chemotherapy, treatment or investigations for infertility, impotence, and operations or medications since the chemotherapy. Patients were also asked about adoption. For the patients still under active follow-up, information from the medical records was used to identify any potential known cause, other than chemotherapy, including prior investigation for infertility, surgery and radiotherapy.

Fertile and infertile GCT patients were compared for age, stage and treatments including cisplatin chemotherapy doses. Significance was assessed by *χ*^2^, Fisher's exact tests and two-tailed *t*-tests as indicated in the results tables using the statistical package SPSS version 10.

In order to compare our results with previously published series, we searched Medline using the search strings GERM CELL TUMOUR, GERM, TERATOMA, INFERTILITY, FERTILITY, CHEMOTHERAPY, AZOOSPERMIA, and CANCER. Bibliographies of relevant studies and previously published reviews were also searched.

## RESULTS

A total of 437 patients (366 men and 71 women) were treated with POMB/ACE for GCTs at Charing Cross Hospital between 1977 and 1996. In total, 335 (279 men and 56 women) were alive and well at the time of the survey. Seven of the women had dysgerminomas, but all the rest had NSGCTs.

### Male GCTs

Questionnaires were sent out to 209 of the 279 treated males, the remainder either being lost to follow-up or excluded because of known infertility as described above in ‘Patients and Methods’. A total of 159 questionnaires were returned (response rate 76%). Of the questionnaires, 153 were assessable, and the mean follow-up period for these patients was 11 years (range 5–24 years). A total of 64 patients (42%) tried to father children after their treatment, of whom 46 (72%) were successful and 18 (28%) were not.

Nonchemotherapy related causes of infertility were identified in 10 of the 18 patients who failed in their attempts to have children (Table 1

**Table 1:** Causes of male infertility in POMB/ACE-treated patients

Total number of patients potentially fertile		153
Total number of patients who tried to have children		64
Total number of patients who were infertile		18
Explained infertility		10
*Radiotherapy*	6	
*Orchiectomy with contralateral atrophic testis*	1	
*Low sperm counts prior to chemotherapy*	2	
*Infertile before chemotherapy but not investigated*	1	
Unexplained infertility		8

). These included radiotherapy (40 Gy in 18 fractions to the para-aortics/pelvic lymph nodes for NSCGTs all treated prior to 1980), atrophic remaining testis, low sperm counts or infertility prior to chemotherapy. Of the remaining eight patients with ‘unexplained infertility’, three had fathered children before receiving chemotherapy, but after treatment were unable to do so due to low sperm counts. It is therefore likely that infertility in these patients was induced by chemotherapy. The remaining five patients failed to father children postchemotherapy but had not had children or sperm counts pretreatment. Interestingly, one of these five patients had a normal sperm count after chemotherapy so failure to have a child may have been due to an infertility problem with his partner. Thus, true chemotherapy-induced infertility only occurred in up to 11% (seven out of 64) of the patients who had attempted to father a child following POMB/ACE treatment.

In total, seven of the 18 infertile patients had children by IVF with donor sperm, and a further two had successful IVF with their own sperm. Only one patient reported temporary difficulty with ejaculation. This patient had not undergone laparotomy or RPLND.

Characteristics of the fertile and infertile groups were compared. There was no significant difference in the average age of the two groups at the time when treatment was started (26.5 and 26.3 years, respectively), or in the proportion of men who had already had children prior to diagnosis (Table 2

**Table 2:** Male age in the fertile and infertile groups at diagnosis: proportion fathering children before diagnosis

	**Fertile *n*=46**	**Not fertile *n*=18**	***P-*value**
Fathered children prior to diagnosis	18 (39%)	4 (22%)	0.2
Didnot father children prior to diagnosis	28 (61%)	14 (78%)	
Age at time of diagnosis	26.5	26.3	0.99

*P*-value determined by *χ*^2^.

). Table 3

**Table 3:** Male infertility rates by stage and treatment

**Patient characteristic**	**% infertile (proportion of total−fertile+infertile)**	***P-*value**
*Stage at diagnosis*		
I	27% (3/11)	
II	28% (7/25)	
III or IV	33% (8/24)	*P*=0.93
Unknown	0% (0/4)	
		
Given radiotherapy	100% (6/6)	*P*<0.0001
No radiotherapy	21% (12/58)	
		
Had a laparotomy/RPLND	43% (6/14)	*P*=0.19
No laparotomy	24% (12/50)	
		
*Including all causes of infertility*		
<400 mg m^−2^ cisplatin	26% (12/46)	
>400 mg m^−2^ cisplatin	29% (5/17)	*P*=0.72
		
*Excluding nonchemotherapy causes of infertility*		
<400 mg m^−2^ cisplatin	13% (5/39)	
>400 mg m^−2^ cisplatin	21% (3/14)	*P*=0.42

*P*-values determined by Fisher's exact or *χ*^2^ tests.

shows that increasing stage did not significantly influence the risk of being infertile. However, dog-leg radiotherapy (4000 rad in 18 fractions) to the para-aortics and pelvic lymph nodes was markedly associated with infertility (*P*<0.0001). In contrast, additional surgery, including laparotomy or RPLND was only associated with a nonsignificant increased risk of infertility (24 *vs* 43% for those not having *vs* undergoing surgery; *P*=0.72).

Since cisplatin has been implicated in the induction of infertility, we examined the mean doses given to the fertile and infertile groups. The fertile group received a mean cisplatin dose of 390 mg m^−2^ and the infertile group 407 mg m^−2^ (*P*=0.65). If the patients with pre-existing infertility, or radiotherapy were excluded, the mean cisplatin doses were 390 mg m^−2^ in the fertile group and 450 mg m^−2^ in the infertile group (*P*=0.24). Previous reports have suggested that patients receiving more than 400 mg m^−2^ of cisplatin are at greater risk of infertility. However, in our series, >400 mg m^−2^ cisplatin did not significantly increase the risk of infertility even if patients who had had radiotherapy or previous low sperm counts were excluded (Table 3). Cyclophosphamide has also been implicated in inducing infertility, particularly at doses above 7500 mg m^−2^. However, Table 4

**Table 4:** Mean doses of chemotherapy in the fertile and infertile groups

	**Fertile**	**Infertile**	**Infertile with no cause apart from chemotherapy**	***P*-value (CI) comparing third with first columns**
Mean dose of cisplatin	390 mg m^−2^ (*n*=45)	407 mg m^−2^ (*n*=18)	450 mg m^−2^ (*n*=8)	*P*=0.24 (−161.4 to 41.3)
Mean dose of cyclo-phosphamide	1500 mg m^−2^ (*n*=45)	1750 mg m^−2^ (*n*=18)	1875 mg m^2^ (*n*=8)	NS

*P*-value determined by *t*-test.

NS=nonsignificant.

shows that the mean dose of cyclophosphamide received was only 1500 mg m^−2^ in the fertile and 1750 mg m^−2^ in the infertile groups (a nonsignificant difference). Consequently, cyclophosphamide may not have contributed to infertility in our patients.

There is clearly a concern that simply having a GCT can contribute to infertility. To try and address this confounding variable, we next examined the risk of infertility in our stage I NSGCT patients who had been placed on surveillance and had never required chemotherapy (Table 5

**Table 5:** Causes of male infertility in nontreated stage 1 patients

Total number of patients potentially fertile		114
Total number of patients who tried to have children		35
Total number of patients who were infertile		9
Explained infertility		6
*Radiotherapy*	3	
*Orchidectomy with contralateral atrophic testis*	1	
*Low sperm counts prior to orchidectomy*	2	
Unexplained infertility		3

). Of the 170 questionnaires sent out, 69% were returned of which 114 were assessable. The mean follow-up for these patients was 10.5 years (range 5–23 years) and was therefore broadly comparable to the POMB/ACE treated patients. In all, 32% (37 out of 114) had no children before their orchidectomy and had not tried to have children afterwards. In all, 37% (42 out of 114) had children before their orchidectomy but had not tried afterwards. Consequently, only 31% (35 out of 114) tried to have children following orchidectomy. Of these, 74% were successful but nine (26%) tried and failed. Potential reasons for failure to have children included radiotherapy (3), known low sperm counts prior to orchidectomy (2) and contra-lateral remaining atrophic testis (1). The remaining three patients had not had children before orchidectomy and have subsequently been unsuccessful having children. One has now stopped trying because his wife has breast cancer and the other two men are currently undergoing investigation with their partners for infertility. Therefore, excluding the men who received radiotherapy, 17% (six out of 35) of stage I NSGCT patients were unable to have children following their orchidectomy.

### Female GCTs

In total, 35 women were alive and well and potentially fertile at the time of the survey, and data were obtained on all of these. The average follow-up time was 13.3 years. Seven of these patients were too young or had not tried to conceive following chemotherapy. Of the 28 remaining patients who tried to become pregnant, 75% (21) succeeded without added intervention, resulting in a total of 36 pregnancies (see Table 6

**Table 6:** Summary of fertility in female GCT patients

Total number of potentially fertile patients	35	
Number who tried to have children	28	
*Number who became pregnant*	21	
Number of pregnancies		36
Number of live births		28
Number of miscarriages		5
Number of terminations		2
Number of malformations		1 ASD
*Number who were infertile*	7	
IVF attempted		2 (1 success)

). A total of 28 pregnancies were carried to term, with only one reported fetal abnormality (an atrial septal defect). There were five miscarriages and two terminations, and one patient was pregnant at the time of the survey. Therefore, 25% of patients (seven out of 28) had an unfulfilled desire for children. However, one of these was already known to have fertility problems and had undergone unsuccessful IVF before chemotherapy. Another had successful IVF following chemotherapy. Of the remaining five patients, three were still having regular periods and two were known to be menopausal postchemotherapy. Consequently, only up to five patients (18%) are likely to have been rendered infertile by the chemotherapy.

The characteristics of the fertile and infertile groups were compared. The mean age of the two groups was equivalent (20 years). There was a small but nonsignificant trend towards higher doses of chemotherapy in the infertile patients. The fertile group received a mean cisplatin dose of 380.9 mg m^−2^ and the infertile group received a mean dose of 424.3 mg m^−2^ (*P*=0.65 95% CI of difference –102.6 to 65.0) (Table 7

**Table 7:** Comparison of the mean cisplatin and cyclophosphamide doses given to women with GCTs who were subsequently found to be fertile or infertile

	**Fertile**	**Infertile**	***P*-value (95% CI)**
Mean dose of cisplatin	380.9^a^ mg m^−2^ (*n*=20)	424.3^a^ mg m^−2^ (*n*=6)	*P*=0.65 (–102.6 to 65.0 mg/m^2^)
Mean dose of cyclophosphamide	1625 mg m^−2^ (*n*=20)	1714 mg m^−2^ (*n*=6)	NS

a One patient who received carboplatin instead of cisplatin was excluded in each group.

*P*-value determined by *t*-test.

NS=nonsignificant.

). In keeping with the male GCT results, there was no significant difference in fertility rates between women receiving above and below 400 mg m^−2^ cisplatin (Table 8

**Table 8:** Comparison of infertility rates in women receiving more or less then 400 mg m^−2^ of cisplatin (*P*=1.00)

	**Fertile**	**Infertile**	**% infertile**	***P*-value**
<400 mg m^−2^ cisplatin	11	3	21%	1.00
>400 mg m^−2^ cisplatin	8	3	27%	

*P* determined by Fisher's exact test.

).

Radiotherapy and laparotomy rates have not been compared for the women, because the numbers of patients are much smaller. One patient received 46 Gy to the pelvis and was not included in the analysis as this dose is almost certain to cause ovarian ablation.

## DISCUSSION

As more patients are cured of their tumours, the long-term consequences of the therapies used become increasingly important. Preservation of fertility is a major issue for patients with GCTs, which are often diagnosed in individuals who have not yet started their families. Previous studies have identified that surgery (RPLND or laparotomy), radiotherapy, bulk of disease and chemotherapy (Rustin *et al*, 1987) can all contribute to subsequent infertility. However, the overall rates of infertility vary widely and can be as high as 60% in men and 40% in women following treatment for their GCTs (see Tables 9

**Table 9:** Published rates of male infertility after treatment for GCTs

**Study**	**Total cases**	**Assessable cases who had chemo**	**Percent seminomas**	**Chemotherapy**	**Number who wished children**	**Fertile patients**	**Number who were infertile (%)**
Roth (Roth *et al*, 1988)	229	147	6	PVB±A^b^	N/A	24	N/A^c^
Hansen (Hansen *et al*, 1990)	177	28	49	Various	8	3	5 (63%)
Bisset (Bissett *et al*, 1990)	120	74	11	Various	N/A	18	N/A
Pont (Pont *et al*, 1996)	42	25	0	2 PEB^a^	N/A	7	N/A
Hartman (Hartmann *et al*, 1999)	124	98	20	Various	40	21	19 (48%)
Petersen (Petersen and Hansen, 1999)	22	22	0	PVB	16	9	7 (44%)
Bohlen (Bohlen *et al*, 2001)	59	49	0	PVB or BEP	N/A	16	5
							
This study	366	153	0	POMB-ACE	64	46	18 (28%)

a PEB/BEP=cisplatin, etoposide, and bleomycin.

b PVB±A=cisplatin, vincristine, and bleomycin with or without adriamycin.

c N/A=data not available from published article.

and 10

**Table 10:** Published rates of female infertility after treatment of GCTs

**Study**	**Total cases**	**Assessable cases who had chemo**	**Percent dysgerminomas**	**Chemotherapy**	**Number who wished children**	**Fertile patients**	**Number who were infertile (%)**
Pektasides (Pektasides *et al*, 1987)	40	17	0%	POMB/ACE	6	5	1 (17%)
Gershenson (Gershenson, 1988)	40	40	N/A^b^	Various	16	11	5 (31%)
Huang (Huang, 1990)	28	26	8%	N/A	10	7	3 (30%)
Wu (Wu *et al*, 1991)	28	22	7%	Various	10	7	3 (30%)
Long (Long *et al*, 1993)	15	15	N/A	N/A	N/A	4	N/A
Mitchell (Mitchell *et al*, 1999)	69	26	0%	Various	N/A	11	N/A
Brewer (Brewer *et al*, 1999)	26	16	100%	BEP^a^	5	3	2 (40%)
Perrin (Perrin *et al*, 1999)	45	29	51%	Various	9	7	2 (22%)
Low (Low *et al*, 2000)	91	39	42%	Various	20	19	1 (5%)
Kanazawa (Kanazawa *et al*, 2000)	31	18	23%	Various	9	8	1 (11%)
Zanetta (Zanetta *et al*, 2001)	169	73	41%	Various	20	16	4 (20%)
Tangir (Tangir *et al*, 2003)	106	64	N/A	Various	38	29	9 (24%)
							
This study	71	35	10%	POMB/ACE	28	21	7 (25%)

a PEB/BEP=Cisplatin, etoposide, and bleomycin.

b N/A=data not available from published article.

). The reasons for this wide variation are likely due to small patient numbers, differences in inclusion/exclusion criteria and the methods used to assess fertility including sperm counts and menstruation *vs* children sired.

To our knowledge, the current study represents one of the largest series concerning infertility postchemotherapy for GCTs. We have found that in those patients who desire a child postchemotherapy, 28% of men and 25% of women have been unsuccessful. Of these, chemotherapy could be implicated as the probable cause in only 11% of men and 18% of women. Approximately 10% of couples in the general population will be infertile (Abma *et al*, 1997). Since patients with GCTs have a higher incidence of infertility than the general population, even before receiving treatment, it is likely that chemotherapy makes little difference to infertility in men and only a marginal difference to women. In keeping with this notion, we found that 17% of male stage I GCT patients were unable to father children despite not having chemotherapy, radiotherapy, or RPLND following their orchidectomy. Moreover, it is of interest that in the only case–control study comparing fertility in stage I male GCT patients who either did or did not receive two cycles of cisplatin containing chemotherapy, there was no excess of infertility in the treated group of patients (Pont *et al*, 1996). Of course, one could argue that the dose of cisplatin received in the Pont study was too low to induce infertility. However, we have not been able to show any dose-dependent correlation with risk of infertility in our study despite the findings of others (Pont and Albrecht, 1997). Nevertheless, we did find that patients receiving above 900 mg m^−2^ of cisplatin lost their fertility (data not shown). Moreover, in women treated for gestational trophoblastic tumours with etoposide, methotrexate, actinomycin D alternating with cyclophosphamide and vincristine (EMA/CO), we similarly failed to find any evidence of reduced fertility postchemotherapy (Woolas *et al*, 1998).

So, why are our findings different from those previously reported? Many of the studies include patients with variable histological types, some of the patient groups are selected for stage of disease, and chemotherapy regimes as well as surgical and radiotherapeutic techniques vary widely. In particular, patients with seminomas are more likely to have received radiotherapy. Some investigators assumed that married patients desired children and unmarried patients did not (Wu *et al*, 1991). In contrast, the data presented here rely almost entirely on fertility rates as reported by patients. However, this is clearly subjective with patient's enthusiasm for filling in and returning questionnaires potentially being affected by their fertility status. In addition, the incidence of nonpaternity is unknown in our male GCT patients. Finally, all studies were observational including our own except Pont *et al* (1996), which was a case–control study.

A number of important questions remain unanswered. New drugs, specifically the taxanes, are being used to treat GCTs (Motzer *et al*, 1994). Very little is known about the long-term effects of these. We mention that seven of the infertile male patients' partners were treated successfully with IVF and donor sperm. In addition, a further two couples were treated successfully with IVF and their own sperm. The impact of new techniques such as intracytoplasmic sperm injection (ICSI) on couples who are sub-fertile after chemotherapy is not yet known. Indeed, the relative success of IVF is not known in couples who have received chemotherapy, although sperm storage prior to treatment is routinely offered.

We conclude that rates of infertility after treatment for GCTs in our series are 28% in men and 25% in women. The contribution of chemotherapy to this is likely to be very small. Moreover, we could not confirm a significant relationship between dose of cisplatin received and chance of infertility. Nevertheless, we still recommend that all male GCT patients are offered sperm storage and when the technology is available, equivalent oocyte storage for female GCT is performed prior to chemotherapy. While there are many theoretical reasons why chemotherapy may induce infertility, it would appear that the risks of this occurring are small.
